# Synthesis and characterization of collagen/PLGA biodegradable skin scaffold fibers

**DOI:** 10.1093/rb/rbx026

**Published:** 2017-09-25

**Authors:** Alireza Sadeghi-Avalshahr, Samira Nokhasteh, Amir Mahdi Molavi, Mohammad Khorsand-Ghayeni, Meysam Mahdavi-Shahri

**Affiliations:** * Materials Research Department, Iranian Academic Center for Education, Culture and Research (ACECR), Khorasan Razavi Branch, Mashhad, Iran

**Keywords:** tissue engineering, composite scaffold, biocompatibility, skin cells

## Abstract

The aim of this study is to investigate the applicability of poly(lactic-co-glycolic acid) (PLGA)/collagen composite scaffold for skin tissue engineering. PLGA and collagen were dissolved in HFIP as a common solvent and fibrous scaffolds were prepared by electrospinning method. The scaffolds were characterized by scanning electron microscopy (SEM), FTIR spectroscopy, mercury porosimetry, tensile strength, biocompatibility assays and Biodegradation. Cytotoxicity and cell adhesion were tested for two cell line groups, human dermal fibroblast (HDF) and human keratinocyte (HaCat). SEM images showed appropriate cell adhesion to the scaffold for both cell lines. MTT assays indicated that the cell viability of HDF cells increased with time, but the number of HaCat cells decreased after 14 days. The ultimate tensile strength was suitable for skin substitute application, but its elongation at break was rather low. For successful clinical application of the PLGA/collagen scaffold, some properties especially mechanical strain needs to be improved.

## Introduction

Collagen is the main structural protein in the extracellular matrix and collagen fibers are capable of accelerating wound healing process [1–5]. Since Collagen is highly hydrophilic, it can improve the interaction of cells with the scaffold. Also, it has the ability to trigger biological signals to support cell adhesion and proliferation [6, 7].

However, these scaffolds suffer from poor mechanical properties for cell morphogenesis. An efficient way to solve this problem is blending this biopolymer with synthetic polymers. When blended with collagen, synthetic polymers can improve the mechanical strength of fibrous scaffold.

Among synthetic polymers, Poly(alpha-hydroxy esters) are biocompatible, biodegradable and possess good mechanical properties. Poly(lactic-co-glycolic acid) (PLGA) is a member of polyesters that has a minimal inflammatory response in the body. The degradation products of PLGA are Lactic acid (LA) and glycolic acid (GA); as a result of ester chains hydrolysis. The degradation rate of PLGA is a function of LA to GA ratio [8, 9].

The ideal skin substitute should imitate the native extra-cellular matrix (ECM) of the body, it’s biological features, and support growth of cells [1, 10]. One of the best scaffold fabrication methods to imitate fibrous structure of skin is electrospinning. Electrospun nanofibers have several advantages such as high specific surface area, high porosity and controllable pore size. These properties can improve cell migration, proliferation and differentiation [3]. However, few research has been done to investigate the ability of PLGA/Collagen electrospun scaffolds for skin tissue engineering applications.

Kwak et al. fabricated multilayer PLGA/collagen using dual extrusion electrospinning technique. The osteoblastic MC3T3-E1proliferated more significantly on mixed fibers compared to pristine PLGA [11]. Considerable proliferation of fibroblast cells has reported due to the high specific surface area and good biological properties of three-dimensional, nanofibrous PLGA/collagen scaffold [12]. In another study a hybrid mesh of web-like collagen microsponges in the openings of a PLGA knitted mesh was prepared. Cell proliferation and extracellular matrix secretion were distributed more uniformly in the hybrid mesh compared to the PLGA scaffold [13]. Wen et al. embedded collagen-grafted PLGA fibers in collagen sponge and used it in liver cell cultivation. Compared to collagen sponge, the compression modulus and hepatocyte cell attachment of hybrid scaffold increased, but collagen sponge showed less cytotoxicity [14].

In this study, PLGA/Type-I collagen fibers were produced by electrospinning. HFIP was used as common solvent. The aim of this research is to provide more information about the properties of this scaffold as skin substitute. In contrast to other investigations, the interaction of the scaffold with two human skin cell groups, fibroblasts and keratinocytes, were separately studied. The fibroblast is the most prevalent cell in dermis that is responsible for synthesizing and deposition of collagen fibers which in turn forms the structural scaffold. Keratinocytes is the principal cell type of the epidermis which grows and stratifies to form a continuous epithelial layer over the surface of the dermal membranes [15, 16]. In addition, in this research the suitability of morphological structure and mechanical properties were evaluated.

## Materials and methods

### Fabrication of composite fibers

The materials used in this study were Type I collagen (Institute Pasteur, Iran), PLGA (90 000–126 000 Da, Sigma-Aldrich) and 1,1,1,3,3-hexafluoro-2-propanol (HFIP, Sigma-Aldrich). PLGA (75:25) flakes and collagen with a ratio of PLGA:Col = 4:1 were dissolved in HFIP as common solvent (20% w/v) and magnetically stirred at room temperature overnight. The polymer solution was then loaded into a syringe, the distance between nozzle tip and collector was set to 17 cm, and then 29 kV voltage was applied to draw out ultra-fine fibers from the spinneret of electrospinning machine (ANSTCo RN/I, Iran). A constant feed rate of 1 ml/h solution was adopted by means of a syringe pump. The electrospun nanofibers were subsequently dried for 24 h in desiccator.

### Material characterizations

To investigate the fiber morphology, the scaffolds were characterized using scanning electron microscope (SEM, LEO-VP 1455). Also the fiber diameters were determined by image J analysis software.

The porosity of PLGA/collagen was measured in 1 mm × 1 mm samples using mercury intrusion porosimeter (PASCAL-140).

FT-IR spectrophotometer (Shimadzu 8400S) was used to record the FT-IR spectra of pure collagen, PLGA and PLGA/collagen fibers. The range of 500–4000 cm^−1^ in transmission mode was employed.

The tensile properties of the electrospun samples with 200 µm thickness (20 mm × 10 mm) were determined by Microtensile testing machine (model TA Plus) equipped with a 50 N load-cell. The crosshead speed was 1 mm/min. Three samples were used for measurement. A similar sample preparation method mentioned by Huang et al. [17]. Also, the ‘wet-state’ mechanical properties of samples were examined immediately after placing the electrospun mats in phosphate buffered saline (PBS) for 24 h at 37 °C. Liquid surface evaporation was negligible during the process.

### Cell culture

HDF human dermal fibroblast and HaCat human keratinocyte cells were cultured in DMEM (Dulbecco’s Modified Eagle Medium) containing 10% fetal bovine serum FBS, 50 U/ml penicillin and 50 U/ml streptomycin. The medium was replaced every 3 days and cultured samples were maintained in a tissue culture incubator at 37 °C with 5% CO_2_. Electrospun mats were sterilized by ultraviolet irradiation for both sides (each side for 20 min); cells were seeded onto scaffolds with density of 2 × 10^4^ cells/cm^2^ and were placed in 12-well plates.

### Cell adhesion

Morphological study of HDF fibroblasts and HaCat keratinocytes grown on electrospun PLGA/Collagen was performed by SEM after 1 day of cell culture. Cell seeded scaffold constructs were washed by PBS and subsequently fixed in 2.5% glutaraldehyde for 3 h.

### Cytotoxicity assay

Cell viability and metabolic activity in response to electrospun samples of PLGA/collagen were measured using MTT (3-[4,5-dimethylthiazol-2-yl]-2, 5 diphenyltetrazolium) assay. Cells were seeded at a density of 5000 cells/cm^2^ and the cell activity was evaluated during a 14-day period. On days 1, 7 and 14, the cell seeded samples were washed with PBS. Then, MTT solution was added to each well. The absorbance was read with an ELISA plate reader at 570 nm.

### Biodegradation

The degradability tests were performed in phosphate buffered saline solution (PBS, pH 7.4) at 37 °C. For each soaking time (1, 7, 14 and 28 days), three samples of PLGA/Collagen mats with 1 cm × 1 cm dimensions were dried and weighed accurately. After soaking time in PBS, the samples were rinsed in distilled water, completely dried and finally weighed. The degradation rate was reported as the weight loss, according to the below formula:

(1) $$\text{weight}-\text{loss}(\%)=\frac{M_{0}-M_{t}}{M_{0}}\times 100,$$

where *M*_0_ and *M*_t_ are sample weights before and after incubation in PBS respectively.

### Statistical analysis

All the data presented are expressed as mean ± standard deviation (SD). Statistical analysis was performed using one-way analysis of variance (ANOVA) with *P*<0.05.

## Results

**Figure 1:**
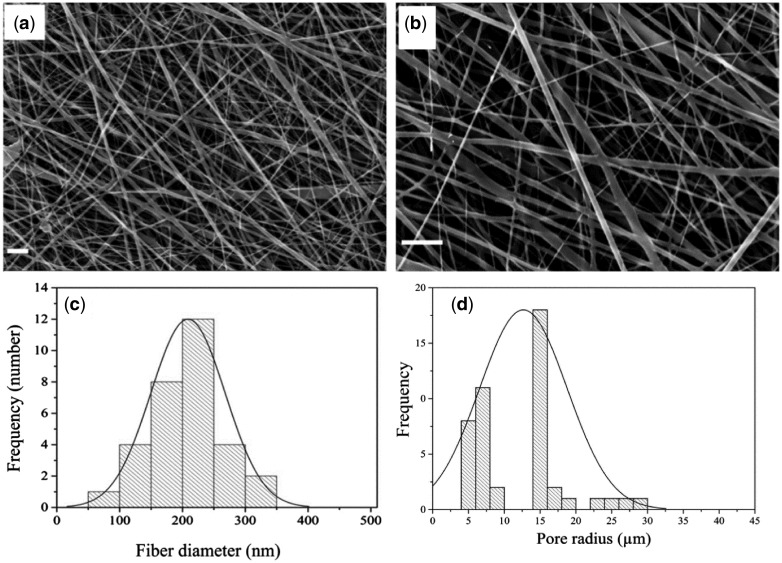
SEM images of collagen/PLGA composite fibers (**a**) 10 000×, (**b**) 20 000×, (scale bar shows 2 μm); (**c**) distribution of fiber diameters; (**d**) porosimetry results of collagen/PLGA

Figure 1a–c shows SEM images of PLGA/collagen fibers. The obtained fibers have no bead and are in the nanometer range. The mean fiber diameter was in the range of 200 ± 60 nm. Also Fig. 1d shows that the pore diameter is in the range of 4–30 µm and the maximum pore size is about 16 µm.

**Figure 2:**
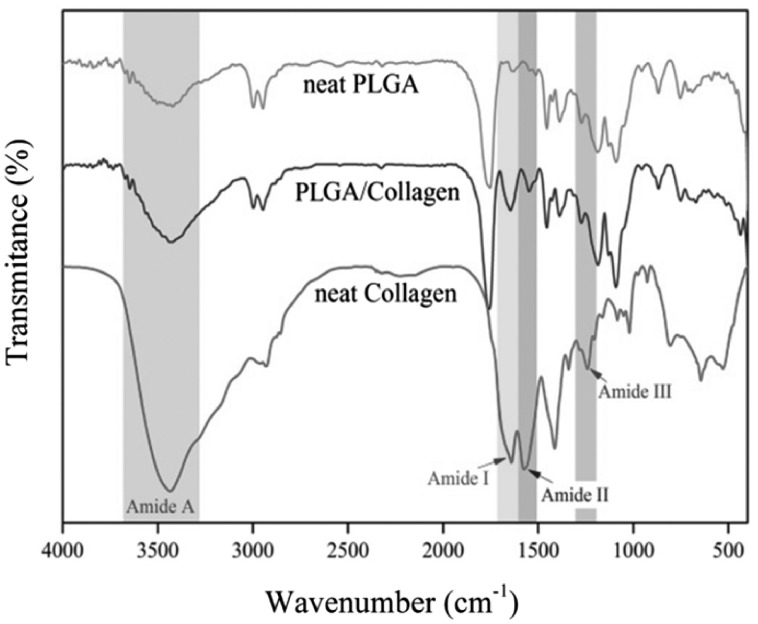
FTIR analysis of collagen, PLGA and PLGA/collagen

FTIR results for collagen, PLGA and composite fibers are shown in Fig. 2. Collagen spectrum depicts characteristic absorption bands at 1640, 1575, 1241 and 34 343 cm^−1^ which represent the amide I, II, III and amide A respectively [18, 19]. PLGA displays characteristic absorption bands at 1100–1250 and 1750–1760 cm^−1^ which represent the esters and carbonyl groups. Also, hydroxyl groups peaks can be observed above 3000 cm^−1^ [19, 20].

**Figure 3:**
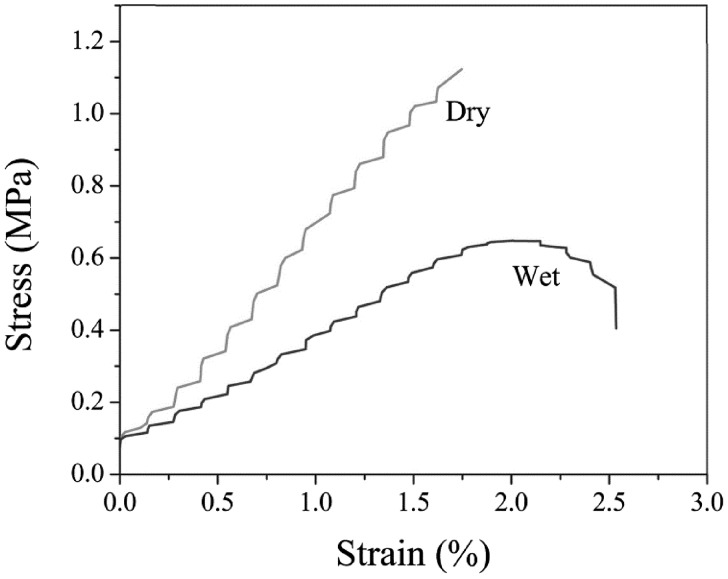
The stress-elongation curve for PLGA/collagen scaffold

Mechanical properties were investigated in dry and wet conditions. As the scaffolds should eventually used *in vivo*, they are evaluated under a simulated physiological environment *in vitro* [21]. The results are shown in Fig. 3. The ultimate tensile strength and the strain were obtained 1.55 ± 0.21 MPa and 1.77 ± 0.41% respectively in dry state; these values changed to 0.65 ± 0.17 MPa and 2.51 ± 0.32% for wet state.

**Figure 4:**
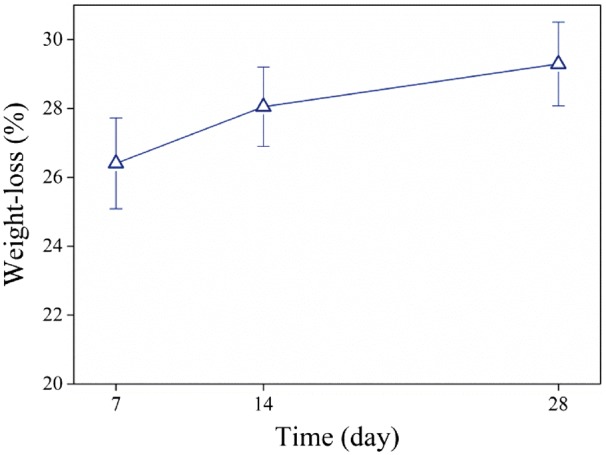
Weight loss of composite fibers

The degradation behavior of the scaffold is shown in Fig. 4. After 4 weeks the scaffold weight loss reached to 29%. The weight loss was relatively high in the first week and then dramatically decreased during the second to fourth week and became almost constant.

**Figure 5:**
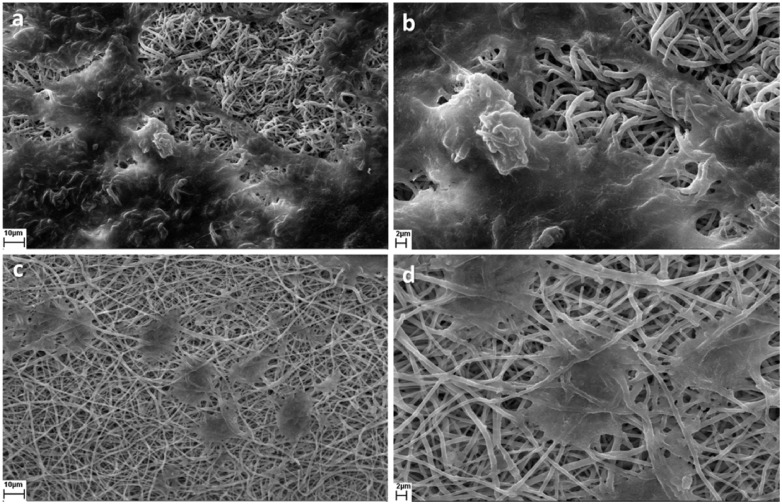
SEM images of cell adhesion (**a, b**) HDF cell line and (**c, d**) HaCat cell line

Figure 5 represents SEM images for fibroblast and keratinocyte cell attachment. Compared to keratinocyte cells, it is obvious that more fibroblast cells have attached to the scaffold and they have speaded better.

**Figure 6:**
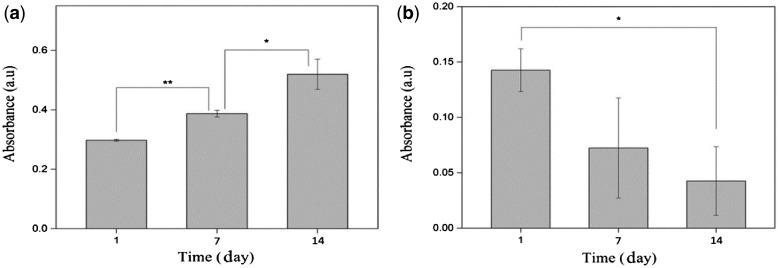
Quantitative evaluation of (**a**) HDF and (**b**) HaCat cytotoxicity (*P < 0.05)

Generally absorption intensity in MTT assay determines the number of viable cells. Viability of HaCat and HDF cells were studied during 14 days. Figure 6a shows that the cultured fibroblast cells on the scaffold have increased over time. Oppositely, the number of viable keratinocyte cells was decreased by increasing time (Fig. 6b).

## Discussion

The aim of this study was to investigate the properties of fibrous PLGA/collagen for application in skin substitute. PLGA was chosen because of its good mechanical properties and collagen was chosen to improve biological properties. Also HFIP was selected as common solvent of both polymers due to its good properties for electrospinning.

The nanometer and bead free fibers (Fig. 1a and b) could be due to the small surface tension of HFIP which is 16 mN/m. Lower electric fields are required for pulling out solution from nozzle when surface tension is low. As a result solution dose doesn′t accumulate at nozzle tip and consequently thinner fibers are obtained. Also High surface tension changes the jet into spheres to make area per mass smaller which produces bead [22]. Figure 2 shows the FTIR spectroscopy results. The characteristic peaks of collagen and PLGA in the FTIR spectra of composite fibers demonstrate the presence of these substances in the sample. Also, the absence of peak shift for both collagen and PLGA along with unchanged relative intensities of PLGA peaks, as the major constituent phase of the scaffold, demonstrates that no interaction has occurred between PLGA and collagen chains.

Stress–strain curves of scaffold are shown in Fig. 3. The percentage of obtained strain is less than natural skin (40–70%). Although collagen constitutes 77% of dermis layer, it has low elongation at break (<10%). Elastin, another component of skin which constitutes 4% of dermis layer, tolerate reversible strain more than 100% before rupture and In fact is responsible for the high strain of skin [23, 24]. Mechanical strength of the scaffold could be suitable for application as skin substitute. Although the obtained strength of the scaffold is far weaker than natural skin, it is comparable with commercial dermal regeneration products such as Integra [25].

Biodegradation of scaffold showed a 29% weight loss after 4 weeks of immersion in PBS. The degradation rate in the first week was very high and then dramatically decreased. As previously mentioned PLGA has a wide range of degradation rate which belongs to LA:GA ratio. As LA amount increases in PLGA, the polymer tends to be more hydrophobic. In a hydrophobic polymer, degradation Usually occurs on the surface and in the hydrophilic one, water enters into the mass and degradation takes place throughout the material [26]. The fast degradation of scaffold is probably due to the hydrophilic nature of collagen that results in a high degradation rate. Considering high molecular weight of used PLGA, the ratio of LA:GA (75:25) which makes it hydrophobic, and the results of other research for low degradation rate of PLGA in the early weeks [27], it seems that in this work collagen plays the major role in the scaffold weight loss.

According to Fig. 5, fibroblast and keratinocyte cells can attach to the scaffold fairly well. Pore size is one of the factors influences cell adhesion. Attachment of cells to large pores is limited because of the big gap to bridge between the pores. The suitable pore diameter for adhesion and growth of cells is normally in the range of 10–100 μm [28]. The obtained porosity in the present study is in the range of 4–30 µm (Fig. 1d), which accords with the above mentioned range and could be a reason for good cell adhesion to the scaffold. Moreover, since collagen is highly hydrophilic, it can improve the interaction of cells with the scaffold. It also has the ability to trigger biological signals to support cell adhesion and proliferation [6, 7]. The suitable properties of collagen originate from its receptors and binding molecules. Cell receptors recognize specific peptide sequence within collagen molecules; this is a direct cell-collagen interaction. Indirect cell-collagen interaction is via binding molecules. There are many RGD-containing proteins which bond to collagen and also recognized by integrin, therefore produce indirect cell-collagen interaction [29].

The absorbance of fibroblast cells increased with time in MTT assay. This represents appropriate cell adhesion and proliferation. In contrast to fibroblasts, the amount of keratinocyte cells decreased with increasing time. Many factors such as constituent materials and microstructure of synthetic scaffolds can influence the proliferation behavior of different cell types. Some cell types are more sensitive to the scaffold characteristics. Comparing to fibroblasts, keratinocyte cells show greater sensitivity to scaffold fiber diameter [30] and presence of GA monomers [31]. Many research has shown that cell proliferation is affected by fiber diameter [32–34]. In addition to this direct effect of fiber diameter, smaller fiber diameter can enhances polymer degradation *in vitro*. This is due to increased surface area which brings about more hydrolysis attack and faster diffusion of the degradation product [35]. Consequently, more LA and GA monomers are produced by reducing fiber diameter. Although these products are non-toxic and can be metabolized in the body, it has shown that high concentrations of GA monomers can inhibit keratinocyte proliferation [31]. Therefore the reduction of viable keratinocyte cells in this research could be attributed to the fiber diameter and of GA monomers. In order to improve biological response, the optimum fiber diameter should be determined.

## Conclusion

Electrospun collagen/PLGA scaffolds were prepared for use in skin tissue engineering. Fiber diameter and pore size distribution were measured 200 ± 60 nm and 4–30 µm respectively. The mechanical strength of the scaffold could be acceptable for skin tissue engineering applications, but the strain was not satisfactory. The scaffold degradation was measured about 29% after 4 weeks, which is suitable for temporary skin substitutes. Cell studies showed that HDF and HaCat cell lines had good surface adhesion on the scaffold. While fibroblasts proliferated with time, the population of keratinocytes decreased. Overall, it seems that mechanical properties and fiber diameter should be modified to make this scaffold applicable as skin substitute.

## Acknowledgment

The authors wish to acknowledge the financial support of the Iranian Academic Center for Education, Culture and Research (ACECR) (Grant No. 2146-21). 

*Conflict of interest statement*. None declared.
